# Tennessee Lunatic Asylum

**Published:** 1841-11

**Authors:** 


### TENNESSEE LUNATIC ASYLUM.

This institution has been in operation now about eighteen months, but from the following extract from the message of the late Governor of the State, its usefulness would appear to be very much limited by the slenderness of the appropriation made for its support:

"In consequence, however, of the limited appropriation made by that act, it has been found impossible to receive several 'insane paupers,' for whose admission into the institution application was made. It is submitted to your consideration whether suitable provision should not be made for the reception and accommodation of all unfortunate persons in the State for whose benefit the institution was established, and for whose admission application may be made.

"A Report will be made to you during your session by the attending physician of the Hospital, who is by law constituted chairman of the Board of Trustees, containing such information, and making such suggestions, in regard to the condition and future management of the institution, as may be deemed useful in your deliberations upon the subject."

We have had opportunities of knowing that the Hospital is very far from being adequate to the demands of that unfortunate class of paupers, for whose benefit it was mainly erected. Great individual suffering, and as great public annoyance are endured in nearly every county in the State, from the insufficiency of the means of the Hospital to receive insane persons who are too poor to pay their expenses in it. This ought not to be. Tennessee is a State of great resources, and great and increasing wealth. Let her representatives, in the Legislature now assembled, be instructed to provide for the clamorous wants of a class of her citizens who cannot take care of themselves. We hope the intelligent physician who has the superintendence of the Hospital, will favor us with a copy of his Report as soon as it is published. Y.

